# Assessment of health care, hospital admissions, and mortality by ethnicity: population-based cohort study of health-system performance in Scotland

**DOI:** 10.1016/S2468-2667(18)30068-9

**Published:** 2018-04-21

**Authors:** Srinivasa Vittal Katikireddi, Genevieve Cezard, Raj S Bhopal, Linda Williams, Anne Douglas, Andrew Millard, Markus Steiner, Duncan Buchanan, Aziz Sheikh, Laurence Gruer

**Affiliations:** a MRC/CSO Social & Public Health Sciences Unit, University of Glasgow, Glasgow, UK; b Edinburgh Migration, Ethnicity and Health Research Group, Centre for Population Health Sciences, Usher Institute for Population Health Sciences and Informatics, University of Edinburgh, Edinburgh, UK; c Population and Health Research Group, School of Geography and Sustainable Development, University of St Andrews, St Andrews, UK; d NHS Health Scotland, Glasgow, UK; e Environmental & Occupational Medicine, Section of Population Health, University of Aberdeen, Aberdeen, UK; f Information Services Division, NHS National Services Scotland, Edinburgh, UK

## Abstract

**Background:**

Ethnic minorities often experience barriers to health care. We studied six established quality indicators of health-system performance across ethnic groups in Scotland.

**Methods:**

In this population-based cohort study, we linked ethnicity from Scotland's Census 2001 (April 29, 2001) to hospital admissions and mortality records, with follow-up until April 30, 2013. Indicators of health-system performance included amenable deaths (ie, deaths avertable by effective treatment), preventable deaths (ie, deaths avertable by public health policy), avoidable deaths (combined amenable and preventable deaths), avoidable hospital admissions, unplanned readmissions, and length of stay. We calculated rate ratios and odds ratios (with 95% CIs) using Poisson and logistic regression, which we multiplied by 100, adjusting first for age-related covariates and then for socioeconomic-related and birthplace-related covariates. The white Scottish population was the reference (rate ratio [RR] 100).

**Findings:**

The results are based on 4·61 million people. During the 50·5 million person-years of study, 1·17 million avoidable hospital admissions, 587 740 unplanned readmissions, and 166 245 avoidable deaths occurred. South Asian groups had higher avoidable hospital admissions than the white Scottish group, with the highest reported RRs in Pakistani groups (RR 140·6 [95% CI 131·9–150·0] in men; RR 141·0 [129·0–154·1] in women). There was little variation between ethnic groups in length of stay or unplanned readmission. Preventable and amenable mortality were higher in the white Scottish group than several ethnic minorities including other white British, other white, Indian, and Chinese groups. Such differences were partly diminished by adjustment for socioeconomic status, whereas adjustment for country of birth had little additional effect.

**Interpretation:**

These data suggest concerns about the access to and quality of primary care to prevent avoidable hospital admissions, especially for south Asians. Relatively high preventable and amenable deaths in white Scottish people, compared with several ethnic minority populations, were unexpected. Future studies should both corroborate and examine explanations for these patterns. Studies using several indicators simultaneously are also required internationally.

**Funding:**

Chief Scientist's Office, Medical Research Council, NHS Research Scotland, Farr Institute.

## Introduction

Differences between ethnic groups have been reported internationally for a range of health outcomes.^1^ Although genetic differences might make a minor contribution, much of this variation is associated with social determinants creating inequalities.^2^ Contributors to such differences include socioeconomic status, health-related behaviours, culture, racism, and health care. When such differences are deemed avoidable and unfair (eg, if they arise through health promotion activities), they can be considered health inequities, which should be addressed.

Universal health coverage aims to provide health care to all, of sufficient quality to be effective, without imposing financial hardship.^3^ However, universal health coverage cannot fully address inequities in health care.^4^ Practices that could result in inequitable health care, though not quantified, include service provision in the dominant language only and dietary advice that is insufficiently adapted to diverse cultures. Culturally competent health care seeks to address such practices. Barriers to accessing effective health care can exacerbate ethnic inequalities in health from other causes, with the delivery of culturally appropriate and accessible health care potentially narrowing inequalities.^5^ Similarly, health gains from public health interventions might differ by ethnicity.^6^

**Research in context**

**Evidence before this study**

We searched MEDLINE and Embase for studies published in English from database inception to Feb 16, 2015, using keywords including “ethnicity”, “race”, “migrants”, “amenable mortality”, “avoidable deaths”, “ambulatory care sensitive conditions”, “avoidable hospitalisations”, and “unplanned readmissions”. We updated this search on Oct 12, 2017. Previous studies were mostly in the USA and New Zealand, with little investigation in European settings. Most studies focused on mortality-based outcomes, finding increased risks of amenable mortality among ethnic minorities. Similarly, studies of avoidable hospital admissions found elevated risks among ethnic or racial minorities. We found no studies that included both primary and secondary care.

**Added value of this study**

Our study investigated ethnic inequalities across a range of dimensions of health-system performance. South Asian groups had relatively high incidence of avoidable hospital admissions compared with the white Scottish ethnic group, suggesting barriers to high quality primary care. Other indicators reflecting quality of hospital care showed less variation, with only slightly increased unplanned readmissions among Pakistani men and women, and no differences in length of stay. By contrast with the existing scientific literature on amenable and preventable mortality, we found little evidence of adverse outcomes in ethnic minorities in Scotland. However, the white Scottish ethnic group had higher preventable and amenable mortality than several ethnic minority groups, with their higher burden partially accounted for by socioeconomic status.

**Implications of all the available evidence**

As reflected in amenable and preventable mortality, there is little evidence of inequitable care for ethnic minorities in NHS Scotland, unlike the few high-income countries that have investigated the topic. For some ethnic groups, especially south Asians, there was evidence that avoidable hospital admissions were relatively high. Our study shows that assessing equity of health system performance across several dimensions simultaneously is feasible using national linked administrative data and is necessary to gain a rounded understanding. We identified some priority areas for improvement—in particular, a greater focus on reducing the high burden of preventable deaths among the white Scottish ethnic group and improved quality of primary care for the Pakistani ethnic group. Future international research on addressing health inequalities by ethnicity, and other aspects of social stratification, should consider studying a range of indicators, rather than relying on mortality outcomes alone. Future research should seek explanations for such patterns, including comorbidities and disease risk factors.

Measuring the performance of health systems is challenging, with a range of indicators used (panel; appendix).^7^ Amenable mortality assesses the quality of a health system by identifying conditions that theoretically should not result in death if timely and effective health care is provided (eg, appendicitis, bacterial meningitis, and ischaemic heart disease).^8^, ^9^ Preventable mortality defines causes of death that can be prevented by effective public health policies (eg, lung cancer and ischaemic heart disease). Deaths can be both amenable and preventable because these categories are not mutually exclusive (appendix). Avoidable mortality combines amenable and preventable deaths.

Panel **Indicators of health-system performance used**

**Mortality indicators**

Mortality-based outcomes classify deaths on the basis of their causes into:

• Amenable deaths, which should not occur if effective health care is received in a timely manner
• Preventable deaths, which should not occur if effective public health measures are implemented
• Avoidable deaths, which could be avoided through full implementation of effective health care and effective public health measures; because some causes of death can be coded under both amenable deaths and preventable deaths, the total number of avoidable deaths is less than the sum of the first two categories

**Primary care indicators**

Hospital admissions that could have been avoided if effective primary care were delivered are referred to as avoidable hospital admissions or, alternatively, ambulatory care-sensitive conditions. These can be subdivided into acute avoidable hospital admissions (related to acute illnesses) and chronic avoidable hospital admissions (related to chronic conditions).

**Secondary care indicators**

The quality of secondary care was assessed primarily by investigating unplanned readmissions, with length of stay also assessed to assess reasons for potential variation in unplanned readmissions.

• Unplanned readmissions: a readmission within 30 days of discharge suggests poor quality hospital care because it could reflect poorly planned discharge processes
• Length of stay: to investigate whether the reason for differences in unplanned readmissions was early discharges among specific ethnic groups, length of stay for selected health conditions was checked to investigate potential early discharge from hospital

The adequacy of primary care has been investigated by identifying causes of hospital admissions that should be prevented by effective and timely primary care (eg, asthma and compulsory psychiatric admission, both conditions with high rates of hospital admission without clear evidence of extra morbidity in the community).^10^ Secondary care quality has been assessed by studying unplanned readmissions and the length of hospital stay.^11^ By convention, readmission within 30 days is the preferred indicator. No standard lengths of stay exist but they can be compared by subgroup. Importantly, comparative assessments across different dimensions of health-system performance remain rare. To minimise deaths, hospital admissions, and readmissions, actions might be required within the health-care system by public health services or policy, or by broader services and policies outside the health system.

Health policy is a devolved responsibility in the four nations comprising the UK.^12^ The UK's National Health Service (NHS) places considerable importance on providing equitable care to all groups, and NHS Scotland has a responsibility to provide an equitable service to all ethnic groups under the Equality Act 2010. However, the contribution of variation in health-care performance on ethnic differences in health outcomes has not previously been investigated in Scotland. We investigated whether delivery of health care and health policy was equitable across ethnic groups by studying quality outcomes based on mortality and hospital admission in the Scottish Health and Ethnicity Linkage Study (SHELS). The health status of the Scottish population has been deteriorating during the past 50 years relative to other nations with similar economies.^13^, ^14^

Scotland is a high-income country providing comprehensive health care free at the point of use for people with residency status, irrespective of ethnic group, country of birth, or nationality. Scotland is characterised by strong political support for ethnic equity from its Government and health agencies.

In general (ie, internationally), ethnic minority groups whether immigrants or offspring fare worse (or are thought to fare worse) in health-care quality and outcomes. In Scotland, the limited quantitative evidence for such a conclusion, mostly from the SHELS, has been conflicting, with the picture varying by outcome and ethnic group. With regard to total mortality, life expectancy, and common cancers, non-white ethnic groups in Scotland are advantaged,^15^, ^16^, ^17^ although Indian and Pakistani populations have more cardiovascular disease than the white Scottish population.^18^ For a wide range of other outcomes, the picture is mixed. National-level, reliable, current data on health-related risk factors are not available in Scotland, placing reliance on the Health Surveys for England 1999 and 2004. The datasets on health-related behaviours that are available, including from Scotland, paint a complex picture (eg, a low prevalence of smoking but a high prevalence of physical inactivity in most non-white ethnic minority groups).^19^

We expected important ethnic group differences in the outcomes reported in this study but did not specify the direction in our analysis plan; however, in view of perceptions about barriers to health care, cultural competence, and the possibility of racism, the performance of the health service would be expected to be suboptimum for non-white ethnic minority populations.

## Methods

### Study design and population

In this population-based cohort study, we assessed three dimensions of health-system performance (mortality-based health outcomes, primary care, and secondary care [panel]) by following a prespecified analysis plan that has also been published online.^16^

The SHELS is a population-based retrospective cohort.^15^, ^20^ In brief, using names, addresses, sex, and dates of birth, Scotland's Census 2001 was securely linked to the Community Health Index, a register of patients using the Scottish NHS. Personal details were replaced by encrypted Community Health Index numbers and encrypted Census numbers that were used to link to Census data, including ethnicity and health outcomes such as death records.

A total of 4·86 million people completed Scotland's Census 2001 (on April 29, 2001), with 4·62 million (95%) successfully linked. The quality of linkage was high, with a false-positive linkage proportion of 0·08%.^21^ The cohort was censored at the time of death, departure from NHS Scotland to other parts of the UK, or the end of the follow-up period (April 30, 2013). The Multi-centre Research Ethics Committee for Scotland and the Privacy Advisory Committee of NHS National Services Scotland gave approval for this study.

### Assessment of ethnicity

Ethnicity was reported in the Census with 14 predefined categories.^20^ One person completes the form on behalf of each person in the household, although optional individual forms for people older than 16 years were available. A high proportion of people in the cohort completed the form (95·7%); we received 100% completeness from the National Records of Scotland who applied their specifically developed imputation methods, using the records of similar people to predict answers.^22^

To minimise the risk of disclosure (based on the rule that a minimum of six events were needed for each category of ethnic group), we combined several categories. We reduced the 14 census categories to ten for the mortality-based outcomes: white Scottish, other white British (primarily English and Welsh), white Irish, other white, any mixed background, Indian, Pakistani, other south Asian (combining Bangladeshi with other south Asian), African origin (combining black African, Caribbean, black Scottish, and other black), and Chinese. The “all other ethnic group” category was removed due to the difficulty of interpreting results for this heterogeneous group.

### Outcomes

We defined amenable, preventable, and avoidable mortality outcomes using the guidance of the Office for National Statistics,^23^ and defined diagnostic codes in death certificates using the tenth revision of the International Statistical Classification of Diseases and Related Health Problems (ICD 10; appendix). Some causes of death are both amenable to medical treatment and preventable through health policy, and these are not mutually exclusive categories; therefore, the number of avoidable deaths is less than the total of preventable and amenable deaths. In view of the large contribution of deaths from ischaemic heart disease, we repeated analyses for amenable mortality with these deaths excluded.

Using the NHS Outcomes Framework,^24^ we coded avoidable hospital admissions into any avoidable hospital admission, avoidable hospital admission for an acute condition, and avoidable hospital admission for a chronic condition (appendix).

We defined unplanned readmissions as being admitted to hospital for non-elective reasons within 30 days of a previous admission.

### Covariates

The National Records Scotland extracted age, sex, country of birth, and indicators of socioeconomic status from the Census. We created a dichotomous variable for country of birth (born in the UK or Ireland *vs* born elsewhere). We used three variables for dimensions of socioeconomic status, which were informed by our method for selecting such variables.^25^ The Scottish Index for Multiple Deprivation is a rank-based measure of deprivation, which is calculated for small areas (known as data zones and comprising a median of 750 individuals). Highest educational attainment typically reflects early adulthood socioeconomic status and was defined on the basis of the individual's highest educational attainment in people aged 16–74 years, and on the basis of the highest educational attainment of the household for the younger and elderly groups (for whom educational status was not collected). The socioeconomic circumstances of a child or elderly person living with a person with higher education were assumed to be better than of those of a child or elderly person who is not living with a person with higher education. Highest educational attainment was categorised as none, low, and high. Household tenure (homeowner *vs* non-homeowner) was used to reflect property-related household wealth.

### Statistical analysis

We followed the prespecified analysis plan with some minor deviation outlined in this paper. Avoidable mortality applied to causes of death at either all ages (eg, HIV/AIDS, complications of the perinatal period, and injuries) or between 0 and 74 years, with the exception of death from diabetes, which applies for those aged 0–49 years. We calculated rate ratios (RRs) with 95% CIs using Poisson regression, with robust variance for the mortality outcomes and with person-years at risk as the denominator. We multiplied RRs by 100 to be interpretable as percentages, as per our analysis plan. The white Scottish ethnic group was the reference category (100). Age-adjusted RRs were calculated by sex. Adjustments for country of birth and socioeconomic status were then added separately and in combination. Socioeconomic status variables were categorical and no specific functional forms were assumed; for example, across the quintile categories of deprivation.

We derived age-adjusted mortality by ethnic group from age-adjusted RRs multiplied by the crude mortality rate of the white Scottish population to calculate the percentage contribution of amenable, preventable, and avoidable deaths to the overall mortality rates.

We used similar analytical approaches for hospital admission-based outcomes. We analysed people aged 19 years and older on the basis of the NHS outcome framework definition for any avoidable hospital admissions, calculating total avoidable hospital admissions during follow-up. We identified each outcome (all, chronic, or acute) from the main hospital discharge diagnosis. The denominator at risk was all hospital admissions during the follow-up (excluding unplanned readmissions), with the numerator being all unplanned readmissions. We used logistic regression because the model was a better fit than Poisson regression.

We analysed length of stay for chronic heart disease, lung cancer, and pneumonia in adults aged 20 years and older, following the SHELS standard age groups. We calculated geometric means for the length of stay in days, comparing ethnic groups using linear regression models of log-transformed lengths of stay. These data were not adjusted for comorbidity because of restrictions on data access.

We analysed data using SAS version 9.4 (SAS Institute Inc, Cary, NC, USA). As required for disclosure control purposes, denominators and numbers of events are presented rounded to the nearest five, but regression models were estimated using exact numbers. Data sharing: the full dataset may be available to researchers via an application to NHS Scotland's Public Benefit and Privacy Panel and to National Records Scotland.

### Role of the funding source

The funders had no role in the study design, data collection, data analysis, data interpretation, or writing of the report. The corresponding author had full access to all intermediate outputs, with the study statisticians (GC, LW, and MS) having access to the full study datasets. All authors had final responsibility for the decision to submit for publication.

## Results

4 607 393 people were included in this analytical sample, of whom 2 409 344 (52·3%) were women (appendix). During 50·5 million person-years of follow-up, 84 705 amenable deaths, 138 065 preventable deaths, 166 245 avoidable deaths, 1·17 million avoidable hospital admissions, and 587 740 unplanned readmissions occurred. Characteristics of the SHELS cohort, including population size by ethnic group, are given in the appendix. In Scotland, in 2001, the other white British, white Irish, and other white minority populations were relatively large, but each non-white group had fewer than 10 000 men or 10 000 women, except for the Pakistani group (which accounted for about 2% of total population). The mean ages of non-white groups were all lower than that of the white Scottish group (39·6 years; 38 years in men and 41 years in women), especially that of the any mixed background group (22·6 years; 21 years in men and 24 years in women). Many non-white people were born in the UK or Ireland (for example, 60% of Pakistani women). Socioeconomic status was highest for the other white British group and varied for non-white ethnic groups, partly dependent on the indicator and sex.

Large ethnic differences were seen for amenable mortality, preventable mortality, and avoidable mortality (table). The white Scottish group had higher age-adjusted amenable mortality than most ethnic minority groups, although rates were imprecisely estimated for some ethnic groups (appendix). The high contribution of preventable deaths to overall mortality in the white Scottish group was substantial (table). By contrast, the lowest contribution of preventable deaths to all-cause mortality, after accounting for age differences, was among Chinese men and women. Although Indian and south Asian men and African-origin and Indian women had a relatively high proportion of preventable deaths, these represented lower absolute burdens of death due to lower all-cause mortality compared with white Scottish people.

**Table:** Amenable, preventable, and avoidable age-adjusted mortality rates per 100 000 person-years and their contribution to all-cause mortality, by sex and ethnic group

	**Total mortality**			**Amenable mortality**		**Preventable mortality**		**Avoidable mortality**	
	Deaths	Person-years	Age-adjusted rate	Age-adjusted rate	Percentage of all deaths	Age-adjusted rate	Percentage of all deaths	Age-adjusted rate	Percentage of all deaths
**Men**									
White Scottish	200 725	21 179 755	947·7	209·6	22·1	359·6	37·9	427·3	45·1
Other white British	12 975	1 571 080	684·8	133·3	19·5	224·1	32·7	265·6	38·8
White Irish	2710	202 190	946·5	205·1	21·7	345·3	36·5	410·4	43·4
Other white	1930	278 515	765·9	146·6	19·1	241·3	31·5	290·0	37·9
Any mixed background	195	56 265	1055·2	211·7	20·1	380·6	36·1	438·4	41·5
Indian	265	65 945	593·3	159·0	26·8	228·6	38·5	259·5	43·7
Pakistani	435	146 430	626·5	195·1	31·1	196·0	31·3	252·3	40·3
Other south Asian	135	35 500	753·7	183·8	24·4	298·9	39·7	348·9	46·3
African origin	130	32 160	846·5	170·2	20·1	259·8	30·7	340·7	40·2
Chinese	195	68 685	495	82·4	16·6	152·1	30·7	188·9	38·2
**Women**									
White Scottish	179 955	22 581 190	796·9	148·9	18·7	228·1	28·6	279·9	35·1
Other white British	11 155	1 644 435	599·5	103·3	17·2	147·3	24·6	180·8	30·2
White Irish	2470	216 905	697·5	123·5	17·7	188·0	27·0	234·1	33·6
Other white	1735	319 915	607·4	108·2	17·8	147·0	24·2	183·7	30·2
Any mixed background	145	59 970	708	129·4	18·3	182·9	25·8	247·7	35·0
Indian	155	59 925	483·5	124·6	25·8	132·1	27·3	161·6	33·4
Pakistani	295	143 940	588·1	130·1	22·1	147·1	25·0	184·6	31·4
Other south Asian	90	28 610	737·8	117·1	15·9	171·4	23·2	204·2	27·7
African origin	90	28 590	648·3	155·7	24·0	186·4	28·8	246·6	38·0
Chinese	175	68 010	524	90·9	17·3	119·3	22·8	164·8	31·5

**Figure 1:**
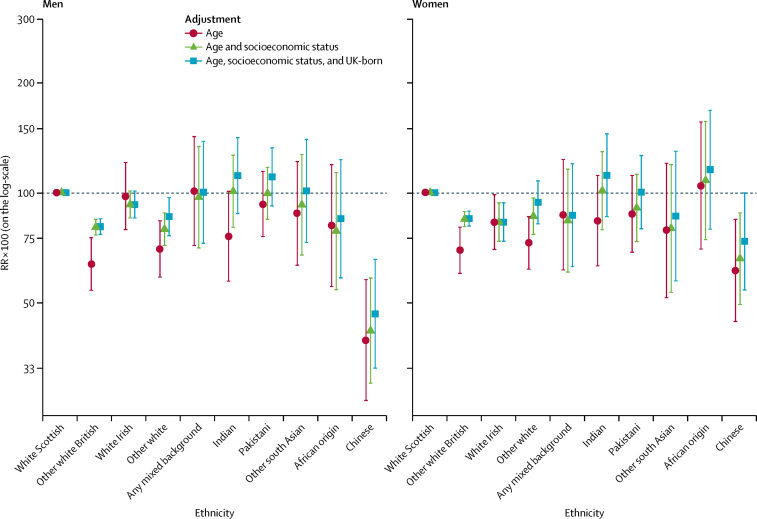
Amenable mortality by ethnic group Shown are RRs multiplied by 100 (so that they can be interpreted as percentage differences between ethnic groups) and adjusted for age; age and socioeconomic status; and age, socioeconomic status, and country of birth. The white Scottish group is the reference category. Bars represent 95% CIs. RR=rate ratio.

In men, the any mixed background (RR 101·0, 95% CI 71·5–142·7) and white Irish (RR 97·9, 79·1–121·1) ethnic groups had similar age-adjusted amenable mortality to the white Scottish (figure 1; appendix); the Chinese (RR 39·3, 26·8–57·7) and other white British (RR 69·9, 58·5–83·6) ethnic groups had the lowest mortality. In women, the Chinese (RR 61·1, 44·2–84·4), other white British (RR 69·4, 59·9–80·3), and other white (RR 72·7, 61·5–85·9) ethnic groups also had lower age-adjusted amenable mortality.

Adjustment for socioeconomic status attenuated, but did not abolish, the difference between the white Scottish and some other ethnic groups (particularly other white British, other white, and Indian) for both men and women. For example, the RR for amenable mortality in other white British men relative to the white Scottish population changed from 63·6 (95% CI 53·9–75·1) to 80·3 (76·4–84·4) after adjustment for socioeconomic status, whereas ad-justment for country of birth resulted in less attenuation (64·0, 54·2–75·6). Adjustment for country of birth moderately attenuated ethnic differences for the other white, Indian, other south Asian, African, Pakistani, and Chinese groups in both sexes.

The findings for amenable mortality, excluding ischaemic heart disease, were broadly similar (appendix). However, the any mixed background ethnic group no longer had high mortality, but this finding was based on few events and was imprecisely estimated.

**Figure 2:**
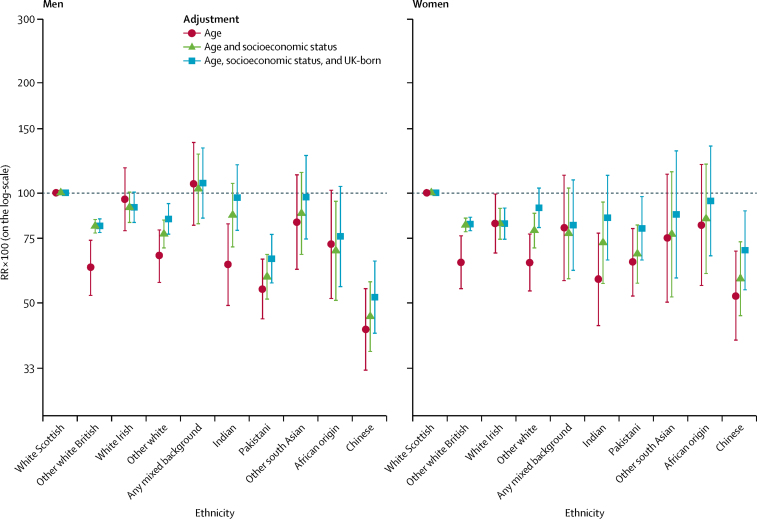
Preventable mortality by ethnic group Shown are RRs multiplied by 100 (so that they can be interpreted as percentage differences between ethnic groups) and adjusted for age; age and socioeconomic status; and age, socioeconomic status, and country of birth. The white Scottish group is the reference category. Bars represent 95% CIs. RR=rate ratio.

The patterns of preventable mortality by ethnic group were similar to those of amenable mortality, although differences tended to be larger (figure 2; appendix). The white Scottish population again had high proportions of age-adjusted preventable mortality in both men (359·6) and women (228·1) per 100 000 person-years, accounting for 37·9% and 28·6% of deaths, respectively, similar to the any mixed background group. The Chinese, Indian, other white, and other white British populations had lower age-adjusted risks than the white Scottish in both sexes. By contrast with amenable mortality, the Pakistani group had much lower preventable mortality than the white Scottish group (RR 54·5 [95% CI 45·2–65·8] in men; RR 64·5 [52·2–79·8] in women). Again, adjustment for socioeconomic status reduced the magnitude of ethnic differences.

**Figure 3:**
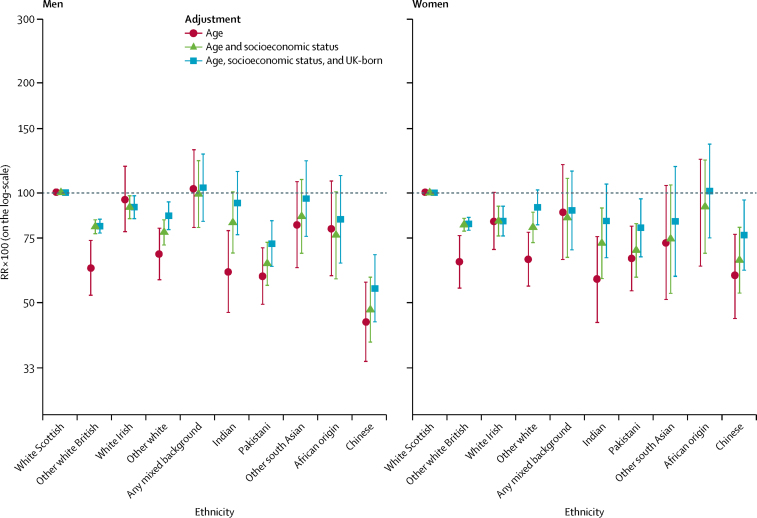
Avoidable mortality by ethnic group Shown are RRs multiplied by 100 (so that they can be interpreted as percentage differences between ethnic groups) and adjusted for age; age and socioeconomic status; and age, socioeconomic status, and country of birth. The white Scottish group is the reference category. Bars represent 95% CIs. RR=rate ratio.

Results for avoidable mortality were similar to those for preventable mortality (figure 3; appendix). In men, overall levels of avoidable mortality were highest among white Scottish, any mixed background, and white Irish groups, with the elevated mortality in white Scottish men partly accounted for by differences in socioeconomic status.

**Figure 4:**
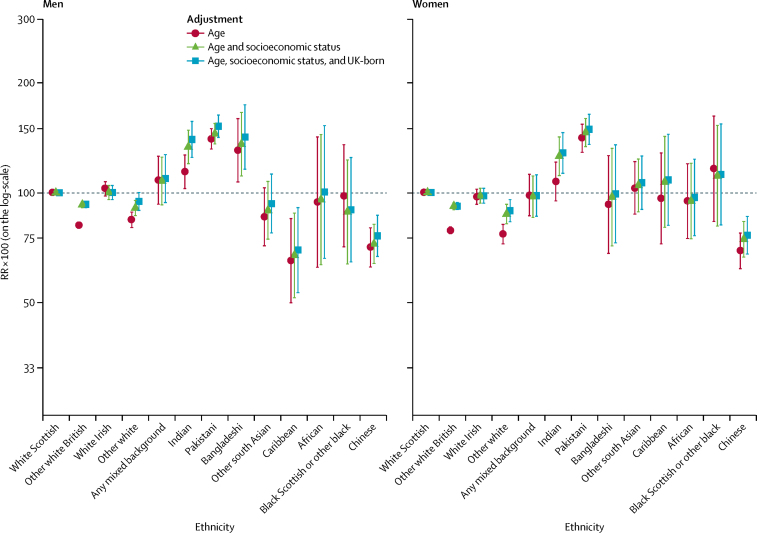
Avoidable hospital admissions by ethnic group Shown are RRs multiplied by 100 (so that they can be interpreted as percentage differences between ethnic groups) and adjusted for age; age and socioeconomic status; and age, socioeconomic status, and country of birth. The white Scottish group is the reference category. Bars represent 95% CIs. RR=rate ratio.

Patterns for avoidable hospital admissions differed from those seen for mortality-based outcomes (figure 4; appendix), with avoidable hospital admissions among some ethnic minority groups much higher than in the white Scottish group. For all-cause avoidable hospital admissions, the Pakistani population had the highest RRs (140·6 [95% CI 131·9–150·0] in men; 141·0 [129·0–154·1] in women), with Bangladeshi and Indian groups also having high RRs in men, but not in women. Adjustment for socioeconomic status tended to increase differences between the white Scottish group and these ethnic minority groups, as did adjustment for country of birth. The other white British and other white ethnic groups had lower avoidable hospital admissions, with adjustment for socioeconomic status attenuating the difference compared with the white Scottish group in both men and women. Caribbean men had lower risk of avoidable hospital admission (RR 65·1, 95% CI 49·9–84·9) than the white Scottish group, whereas Caribbean women did not (RR 96·3, 72·2–128·4). The RRs for the Chinese ethnic group were consistently low, too. Similar ethnic patterns were seen for both acute and chronic avoidable hospital admissions, although ethnic differences were larger for chronic avoidable hospital admissions (appendix).

Differences in unplanned readmissions were slight (appendix). In men, the Pakistani ethnic group had higher odds ratios (ORs) for unplanned readmissions (OR 113·8, 95% CI 104·0–124·6), as did the Indian ethnic group after adjustment for covariates. Pakistani women also had slightly higher ORs after adjustment. The other white British group had lower ORs for unplanned readmission in both men and women, but adjustment for socioeconomic status substantially narrowed the difference with the white Scottish comparison group (in men, age-adjusted OR 86·0 [95% CI 83·4–88·7] *vs* age and socioeconomic status-adjusted OR 95·3 [92·5–98·1]; in women, age-adjusted OR 89·7 [87·2–92·2] *vs* age and socioeconomic status-adjusted OR 97·3 [94·6–100·0]).

Length of stay for hospital admissions due to three diagnoses showed no consistent differences between ethnic groups (appendix).

## Discussion

Our study is a nationwide assessment using a set of measures of health-system performance by ethnicity. We found no evidence of systematically poorer health outcomes across ethnic minority groups in Scotland. Instead, we found that the white Scottish ethnic group had a relatively high burden of amenable and preventable deaths compared with other ethnic groups, possibly partly related to poorer socioeconomic circumstances. The findings on avoidable hospital admissions suggest that some south Asian ethnic groups need improved access to primary care or improved quality of primary care, or both. Further investigation of the variations should provide insights to support a policy response.

A barrier to investigating how well health systems meet the needs of ethnic minorities has been the paucity of data for quantitative analysis, with ethnicity recording being poor in many countries.^26^ Most such research has been in the USA and New Zealand.^27^ In the USA, where ethnicity and race are more strongly related to socioeconomic circumstances than in the UK, studies have found higher amenable mortality among black than white populations.^28^, ^29^ Similarly, ethnic and racial minorities in the USA have been found to have an increased risk of ambulatory care-sensitive conditions^30^, ^31^ and lower levels of receipt of high-quality treatment white ethnic groups.^32^ In New Zealand, amenable mortality makes an important contribution to the greater mortality risk among the Pacific peoples when compared with the European/other reference population (accounting for 26·2% of the disparity in men and 33·8% in women in 2001–04, unlike the opposite pattern of findings reported in our study).^33^, ^34^ Important variations in amenable mortality have also been reported in Singapore, a country with three major ethnic groups.^35^ The evidence is especially scarce within Europe. A 2017 systematic review^27^ found no studies of avoidable hospital admissions within Europe by ethnicity, race, or migration status, and only three longitudinal studies worldwide. A study by de Bruijne and colleagues,^11^ which found ethnic variations in unplanned readmissions and excess length of stay in the Netherlands, seems to be the only study to investigate the quality of health-care delivery by ethnicity in Europe. By contrast with our study, the authors suggest that shortcomings in the quality of the hospital care exist among people who were not born in western countries who have moved to the Netherlands.

Our study has several strengths, including simultaneous assessment of six dimensions of health-system performance in primary and secondary care. Comparison of patterns across these has indicated how complex issues are and the challenges for articulating health policy to respond to ethnic inequalities. We studied virtually the whole population, thereby minimising the non-response that occurs when using survey-based data. The private hospital sector is small in Scotland and it is required to supply data to the Information Services Division. Our study has allowed heterogeneity among usually broadly defined ethnic groups, such as within the south Asian group, to be investigated.

This study has some limitations. Although the indicators in this study are widely used, their validity remains unclear and they might be better viewed as an indicator for further investigation being needed.^7^, ^36^ The Office for National Statistics classification we used is not the only one, and has the drawback that the categories of amenable and preventable deaths are not mutually exclusive.^37^ Other potential indicators of health service performance exist but have not been included in this study (eg, quality of life, disability, and aspects of morbidity). Although our study is large, the number of events within some ethnic groups is small and some important ethnic inequalities might have been missed. Further assessment of the any mixed background ethnic group would be particularly important. Adjustment for further socioeconomic covariates (such as occupation^38^) could result in slightly differing findings, but given our structured approach to identifying appropriate socioeconomic covariates,^25^ our main results are unlikely to change. We did not adjust for disease risk factors (such as hypertension, obesity, and smoking) because of a scarcity of such data on a national scale in Scotland.^18^ Ideally, such adjustments will be done in the future. We did not examine the results for those born in and outside the UK separately because of insufficient statistical power, but would recommend such research for the future once the number of outcomes has increased over time and greater statistical power is available. Some people living in Scotland in 2001 were not linked to our database and some would have outcomes outside Scotland (eg, death abroad following travel or re-emigration). We cannot quantify the effects of incomplete data. We did not examine readmission by diagnosis. The generalisability of our results to the rest of the world is unclear. In view of changes in Scotland's ethnic group composition since 2001, this research warrants replication using linkage to the 2011 Census.

The UK has performed poorly when compared with similar high-income countries in improving premature mortality.^39^ We add an important equity dimension to that analysis. On the basis of the limited available scientific literature, NHS Scotland seems to provide more equitable care for ethnic minorities than other high-income countries, which have attempted to assess the equity of health-system performance by ethnicity. Scotland's focus on ethnic equity within health policy might hold clues to realising improvements elsewhere. Our study suggests areas for improvement. A focus on improving the quality of primary care for ethnic minority groups (particularly south Asians) is warranted, especially because previous studies suggest suboptimum primary care might contribute to poor asthma and mental health outcomes.^40^, ^41^ In the context of long-term conditions such as asthma, there is a need to ensure appropriate self-management education, highlight the importance of regular use of preventive treatments, and ensure that individuals can detect early signs of a deterioration in their clinical condition and then know how to respond effectively.^42^ Improvements in diabetes care might be similarly important.^43^

Our study emphasises the importance of studying several dimensions of health-system and health-policy performance in view of differences in the patterns shown by ethnic group (eg, between avoidable mortality and hospital admission). The complexity inherent in these patterns has also been found in other outcomes studied in SHELS. For example, excepting the Chinese population in whom health is generally good, some health outcomes are better and some worse in ethnic minority groups than in the reference white Scottish group (eg, cancers tend to be low compared with the reference group in most ethnic minority groups but cardiovascular diseases are commoner in south Asian groups).^15^, ^21^ White Scottish populations fare worse in several outcomes but have the highest level of breast cancer screening, an indicator of health care.^44^ Generalisations are difficult and evaluations need to be made for each outcome and for each ethnic group. This principle will likely apply outside of Scotland. The high preventable mortality among the white Scottish group requires a policy response that extends beyond the NHS to improve the social determinants of health, including health-related risk factors such as alcohol, tobacco, and poor diet. Such analysis benefits from examination through the lens of ethnic variations.
