# Supplementary material for: Assessment of health care, hospital admissions, and mortality by ethnicity: population-based cohort study of health-system performance in Scotland
Source: Lancet Public Health. 2018 Apr 21;3(5):e226–36. doi: 10.1016/S2468-2667(18)30068-9 (PMC5937910; doi:10.1016/S2468-2667(18)30068-9)
Supplement: Supplementary appendix [file mmc1.pdf]

THE LANCET
Public Health

Supplementary appendix

This appendix formed part of the original submission and has been peer reviewed. We post it as supplied by the authors.

Supplement to: Katikireddi SV, Cezard G, Bhopal RS, et al. Assessment of health care, hospital admissions, and mortality by ethnicity: population-based cohort study of health-system performance in Scotland. *Lancet Public Health* 2018; published online April 20. http://dx.doi.org/10.1016/S2468-2667(18)30068-9.

<u>Web Appendix: Assessing equity of healthcare in ethnic minorities</u>

Contents

1. Categorisation of amenable, preventable and avoidable causes of death
2. Categorisation of avoidable hospitalisations
3. Socio-demographic characteristics of analytical sample
4. Amenable mortality results
5. Amenable mortality (excluding IHD) results
6. Preventable mortality results
7. Avoidable mortality results
8. All avoidable hospitalisations results
9. Acute avoidable hospitalisations results
10. Chronic avoidable hospitalisations results
11. Unplanned readmissions results
12. Length of stay results

Table 1: Causes of death (classified using the International Classification of Diseases, tenth revision) considered to be avoidable

Avoidable deaths are those that are either amenable or preventable (all those listed below). Classification is based on underlying cause of death.

Condition group and cause	ICD-10 codes	Age	Amenable	Preventable
**Infections**				
Tuberculosis	A15-A19, B90	0-74	•	•
Selected invasive bacterial and protozoal infections	A38-A41, A46, A48.1, B50-B54, G00, G03, J02, L03	0-74	•	
Hepatitis C	B17.1, B18.2	0-74	•	•
HIV/AIDS	B20-B24	All	•	•
**Neoplasms**				
Malignant neoplasm of lip, oral cavity and pharynx	C00-C14	0-74		•
Malignant neoplasm of oesophagus	C15	0-74		•
Malignant neoplasm of stomach	C16	0-74		•
Malignant neoplasm of colon and rectum	C18-C21	0-74	•	•
Malignant neoplasm of liver	C22	0-74		•
Malignant neoplasm of trachea, bronchus and lung	C33-C34	0-74		•
Malignant melanoma of skin	C43	0-74	•	•
Mesothelioma	C45	0-74		•
Malignant neoplasm of breast	C50	0-74	•	•
Malignant neoplasm of cervix uteri	C53	0-74	•	•
Malignant neoplasm of bladder	C67	0-74	•	
Malignant neoplasm of thyroid gland	C73	0-74	•	

Hodgkin's disease	C81	0-74	•	
Leukaemia	C91, C92.0	0-44	•	
Benign neoplasms	D10-D36	0-74	•	
**Nutritional, endocrine and metabolic**				
Diabetes mellitus	E10-E14	0-49	•	•
**Drug use disorders**				
Alcohol related diseases, excluding external causes	F10, G31.2, G62.1, I42.6, K29.2, K70, K73, K74 (excl. K74.3-K74.5), K86.0	0-74		•
Illicit drug use disorders	F11-F16, F18-F19	0-74		•
**Neurological disorders**				
Epilepsy and status epilepticus	G40-G41	0-74	•	
**Cardiovascular diseases**				
Rheumatic and other valvular heart disease	I01-I09	0-74	•	
Hypertensive diseases	I10-I15	0-74	•	
Ischaemic heart disease	I20-I25	0-74	•	•
DVT with pulmonary embolism	I26, I80.1-I80.3, I80.9, I82.9	0-74		•
Cerebrovascular diseases	I60-I69	0-74	•	
Aortic aneurysm and dissection	I71	0-74		•
**Respiratory diseases**				
Influenza (including swine flu)	J09-J11	0-74	•	•
Pneumonia	J12-J18	0-74	•	
Chronic obstructive pulmonary disorder	J40-J44	0-74		•
Asthma	J45-J46	0-74	•	
**Digestive disorders**				
Gastric and duodenal ulcer	K25-K28	0-74	•	

Acute abdomen, appendicitis, intestinal obstruction, cholecystitis/lithiasis, pancreatitis, hernia	K35-K38, K40-K46, K80-K83, K85, K86.1-K86.9, K91.5	0-74	•	
**Genitourinary disorders**				
Nephritis and nephrosis	N00-N07, N17-N19, N25-N27	0-74	•	
Obstructive uropathy and prostatic hyperplasia	N13, N20-N21, N35, N40, N99.1	0-74	•	
**Maternal and infant**				
Complications of perinatal period	P00-P96, A33	All	•	
Congenital malformations, deformations and chromosomal anomalies	Q00-Q99	0-74	•	
**Unintentional injuries**				
Transport Accidents	V01-V99	All		•
Accidental Injury	W00-X59	All		•
**Intentional injuries**				
Suicide and self inflicted injuries	X60-X84, Y10-Y34	All		•
Homicide/Assault	X85-Y09, U50.9	All		•
Misadventures to patients during surgical and medical care	Y60-Y69, Y83-Y84	All	•	•

Box 1: Categorisation of ischaemic heart disease within mortality and hospitalisation-based outcomes

Ischaemic heart disease (ICD code I 20-I 25): an example of a condition that is considered amenable and preventable mortality at ages 0-74 years, and hence a contributor to avoidable mortality and avoidable hospitalisation/rehospitalisation. Within the UK, these definitions are used for monitoring and evaluating health policy and therefore have considerable policy relevance.

The major causes of ischaemic heart disease are well known and are changeable through personal, community level and societal interventions. At least 70% of this disease is thought to be theoretically preventable through actions such as stopping smoking, reducing LDL cholesterol levels through dietary and other changes, reduction of blood pressure through actions including reducing salt intake, and increasing physical activity.

Currently heart disease is also amenable to health care interventions e.g. successful treatment of cardiac arrest, thrombolysis of blood clots, and long-term action such as cardiac rehabilitation, and taking of drugs including statins. The actions for prevention will also be helpful for reducing the risk of a second ischaemic event.

Together, these kinds of interventions mean that ischaemic heart disease is largely avoidable. This condition exemplifies that the figures for avoidable mortality include both interventions that overlap in regards to preventability and amenability.

Given its preventability, and the opportunity to prevent new events, some specific conditions within the category ischaemic heart disease (ICD 10 codes I 24.8, I 24.9, and I 25) contribute to avoidable hospitalisation. Rehospitalisation within 30 days of discharge with a diagnosis of ischaemic heart disease would likely indicate suboptimal management of the original reason for hospitalisation.

Table 2: Hospitalisation codes considered to be avoidable (NHS Outcomes Framework)

ICD10 codes	Condition	Acute	Chronic
A02.0	Salmonella enteritis	X	
A04	Other bacterial intestinal infections	X	
A05.9	Bacterial foodborne intoxication, unspecified	X	
A07.2	Cryptosporidiosis	X	
A08	Viral and other specified intenstinal infections	X	
A09	Diarrhoea and gastroenteritis of presumed infectious origin	X	
A36	Diphtheria	X	
A37	Whooping cough	X	
A69.0	Necrotizing ulcerative stomatitis	X	
B05	Measles	X	
B06	Rubella	X	
B16.1	Acute hepatitis B with delta-agent without hepatic coma	X	
B16.9	Acute hepatitis B without delta-agent and without hepatic coma	X	
B18.0	Chronic viral hepatitis B with delta-agent		X
B18.1	Chronic viral hepatitis B without delta-agent		X
B26	Mumps	X	
D50.1	Sideropenic dysphagia		X
D50.8	Other iron deficiency anemias		X
D50.9	Iron deficiency anemia, unspecified		X
D51	Vitamin B12 deficiency anaemia		X
D52	Folate deficiency anaemia		X
E10	Type 1 diabetes mellitus		X
E11	Type 2 diabetes mellitus		X
E12	Malnutrition-related diabetes mellitus		X
E13	Other specified diabetes mellitus		X
E14	Unspecified diabetes mellitus		X
E86	Volume depletion	X	
F00	Dementia in alzheimers		X
F01	Vascular dementia		X
F02	Dementia in other diseases		X
F03	Unspecified dementia		X
G25.3	Myoclonus	X	
G40	Epilepsy and recurrent seizures		X
G41	Status epilepticus		X
H66	Suppurative and unspecified otitis media	X	
H67	Otitis media in diseases classified elsewhere	X	
I10X	Essential (primary) hypertension		X
I11.0	Hypertensive heart disease with heart failure		X
I11.9	Hypertensive heart disease without heart failure		X
I13.0	Hypertensive heart and renal disease with (congestive) heart failure		X
I20	Angina pectoris		X
I24.0	Coronary thrombosis not resulting in myocardial infarction	X	
I24.8	Other forms of acute ischaemic heart disease	X	
I24.9	Acute ischaemic heart disease, unspecified	X	
I25	Chronic ischaemic heart disease		X
I48X	Atrial fibrillation and flutter		X

I50	Heart failure		X
I89.1	Lymphangitis	X	
J02	Acute pharyngitis	X	
J03	Acute tonsillitis	X	
J04.0	Acute laryngitis	X	
J06	Acute upper respiratory infections multiple and unsp sites	X	
J10	Influenza due to other identified influenza virus	X	
J11	Influenza due to unidentified influenza virus	X	
J13X	Pneumonia due to Streptococcus pneumoniae	X	
J14	Pneumonia due to Hemophilus influenzae	X	
J15.3	Pneumonia due to streptococcus, group B	X	
J15.4	Pneumonia due to other streptococci	X	
J15.7	Pneumonia due to Mycoplasma pneumoniae	X	
J15.9	Unspecified bacterial pneumonia	X	
J16.8	Pneumonia due to other specified infectious organisms	X	
J18.1	Lobar pneumonia, unspecified organism	X	
J18.8	Other pneumonia, unspecified organism	X	
J20	Acute bronchitis		X
J31.2	Chronic pharyngitis	X	
J41	Simple and mucopurulent chronic bronchitis		X
J42X	Unspecified chronic bronchitis		X
J43	Emphysema		X
J44	Other chronic obstructive pulmonary disease		X
J45	Asthma		X
J46X	Status asthmaticus		X
J47X	Bronchiectasis		X
J81X	Pulmonary edema		X
K02	Dental caries	X	
K03	Other diseases of hard tissues of teeth	X	
K04	Diseases of pulp and periapical tissues	X	
K05	Gingivitis and periodontal diseases	X	
K06	Other disorders of gingiva and edentulous alveolar ridge	X	
K08	Other disorders of teeth and supporting structures	X	
K09.8	Other cysts of oral region, not elsewhere classified	X	
K09.9	Cyst of oral region, unspecified	X	
K12	Stomatitis and related lesions	X	
K13	Other diseases of lip and oral mucosa	X	
K20	Esophagitis	X	
K21	Gastro-oesophageal reflux disease	X	
K25.0	Acute gastric ulcer with hemorrhage	X	
K25.1	Acute gastric ulcer with perforation	X	
K25.2	Acute gastric ulcer with both hemorrhage and perforation	X	
K25.4	Chronic or unspecified gastric ulcer with hemorrhage	X	
K25.5	Chronic or unspecified gastric ulcer with perforation	X	
K25.6	Chronic or unspecified gastric ulcer with both hemorrhage and perforation	X	
K26.0	Acute duodenal ulcer with hemorrhage	X	
K26.1	Acute duodenal ulcer with perforation	X	
K26.2	Acute duodenal ulcer with both hemorrhage and perforation	X	
K26.4	Chronic or unspecified duodenal ulcer with hemorrhage	X	
K26.5	Chronic or unspecified duodenal ulcer with perforation	X	
K26.6	Chronic or unspecified duodenal ulcer with both	X	

	hemorrhage and perforation		
K27.0	Acute peptic ulcer, site unspecified, with hemorrhage	X	
K27.1	Acute peptic ulcer, site unspecified, with perforation	X	
K27.2	Acute peptic ulcer, site unspecified, with both hemorrhage and perforation	X	
K27.4	Chronic or unspecified peptic ulcer, site unspecified, with hemorrhage	X	
K27.5	Chronic or unspecified peptic ulcer, site unspecified, with perforation	X	
K27.6	Chronic or unspecified peptic ulcer, site unspecified, with both hemorrhage and perforation	X	
K28.0	Acute gastrojejunal ulcer with hemorrhage	X	
K28.1	Acute gastrojejunal ulcer with perforation	X	
K28.2	Acute gastrojejunal ulcer with both hemorrhage and perforation	X	
K28.4	Chronic or unspecified gastrojejunal ulcer with hemorrhage	X	
K28.5	Chronic or unspecified gastrojejunal ulcer with perforation	X	
K28.6	Chronic or unspecified gastrojejunal ulcer with both hemorrhage and perforation	X	
K52	Other noninfective gastroenteritis and colitis	X	
L01	Impetigo	X	
L02	Cutaneous abscess, furuncle and carbuncle	X	
L03	Cellulitis	X	
L04	Acute lymphadenitis	X	
L08.0	Pyoderma	X	
L08.8	Other specified local infections of skin and subcutaneous tissue	X	
L08.9	Local infection of skin and subcutaneous tissue, unspecified	X	
L88	Pyoderma gangrenosum	X	
L98.0	Pyogenic granuloma	X	
M01.4	Rubella arthritis	X	
N10	Acute tubulo-interstitial nephritis	X	
N11	Chronic tubulo-interstitial nephritis	X	
N12	Tubulo-interstitial nephritis not specified as acute or chronic	X	
N13.6	Pyonephrosis	X	
N15.9	Renal tubulo-interstitial disease, unspecified	X	
N30.0	Acute cystitis	X	
N30.8	Other cystitis	X	
N30.9	Cystitis, unspecified	X	
N39.0	Urinary tract infection, site not specified	X	
O15	Eclampsia	X	
R56	Convulsions, not elsewhere classified	X	

Table 3: Socio-demographic characteristics of the linked Census population by sex

Sex and ethnic group	N	Age at Census in years		Country of Birth	SIMD		Highest Qualification (individual)			Highest Qualification (household)			Household Tenure	
		(mean, SD)		*UK born (%)*	*Most Deprived (%)*	*Least Deprived (%)*	*No*	*Low*	*High*	*No*	*Low*	*High*	*Rented*	*Owned*
**Males**														
White Scottish	1949484	38	(22)	99.1	20.1	19.8	34.2	41.4	24.4	20.2	40.8	39.0	31.4	68.6
Other White British	160235	42	(20)	95.3	8.0	29.6	17.2	34.5	48.4	10.0	28.1	61.9	27.7	72.3
White Irish	20341	45	(20)	98.4	22.2	21.7	32.7	30.5	36.8	19.8	28.8	51.4	34.6	65.4
Other White	29944	36	(21)	30.5	12.4	32.6	24.4	26.5	49.2	11.5	22.3	66.2	43.4	56.6
Any Mixed Background	5310	21	(18)	76.0	19.7	26.1	21.1	42.1	36.8	12.0	28.3	59.7	42.9	57.1
Indian	6448	31	(19)	48.4	9.7	38.5	21.5	27.6	50.9	10.0	25.5	64.5	27.8	72.2
Pakistani	12929	27	(19)	58.0	15.8	24.8	39.7	33.9	26.5	17.3	35.2	47.5	23.8	76.2
Other South Asian	3549	29	(19)	38.9	24.2	28.3	25.3	28.3	46.4	15.9	25.4	58.7	46.3	53.7
African origin	3277	30	(18)	39.9	27.6	22.4	17.3	30.3	52.4	9.9	21.9	68.2	56.9	43.1
Chinese	6532	30	(18)	38.4	13.4	38.5	39.0	29.2	31.8	21.2	30.5	48.3	30.2	69.8
**Females**														
White Scottish	2138643	41	(24)	99.1	21.3	19.2	35.7	40.6	23.8	21.9	40.3	37.8	34.8	65.2
Other White British	174748	44	(21)	95.0	8.2	28.9	19.8	39.4	40.8	11.5	30.0	58.5	30.5	69.5
White Irish	23162	49	(21)	98.6	20.1	22.8	32.9	29.1	38.0	20.8	25.8	53.4	35.9	64.1
Other White	35711	37	(21)	26.4	10.8	33.4	21.5	27.2	51.3	10.7	20.7	68.7	41.3	58.7
Any Mixed Background	5799	24	(20)	74.5	18.7	27.3	19.9	42.3	37.8	11.5	28.3	60.2	44.2	55.8
Indian	5888	30	(19)	51.1	9.6	39.0	27.0	32.3	40.7	10.8	27.3	61.9	26.4	73.6
Pakistani	12702	26	(18)	60.5	15.4	24.6	44.3	32.9	22.8	17.1	35.8	47.1	24.1	75.9
Other South Asian	2963	29	(20)	44.5	22.0	28.9	29.5	33.7	36.7	18.0	26.9	55.2	45.5	54.5
African origin	3056	30	(18)	42.1	27.5	24.5	17.8	36.1	46.1	10.2	22.2	67.6	54.9	45.1
Chinese	6672	31	(18)	33.9	12.1	39.1	37.6	29.0	33.3	20.1	29.5	50.3	29.9	70.1

Table 4: Age adjusted rates per 100,000 population (PY) and rate ratios (RR x100) for amenable mortality by sex and ethnic group. RRs are age, socio-economic status (SES) (household tenure, combined individual and household education and Scottish Index of Multiple Deprivation SIMD) and UK/RoI-born (versus born elsewhere) adjusted, with 95% confidence intervals.

Ethnic group	Any amenable death	PY at risk	Age-adjusted poisson rates (for 100,000 PY)	Age adjusted RR and 95% CI	Age and SES adjusted RR and 95% CI	Age and UK/RoI-born adjusted RR and 95% CI	Age, UK/RoI-born and SES adjusted RR and 95% CI
MALES							
White Scottish	44390	21179755	209.6	100.0	100.0	100.0	100.0
Other White British	2570	1571080	133.3	63.6 (53.9, 75.1)	80.3 (76.4, 84.4)	64 (54.2, 75.6)	80.6 (76.7, 84.7)
White Irish	550	202190	205.1	97.9 (79.1, 121.1)	92.7 (85.1, 101)	97.9 (79.1, 121.2)	92.7 (85.1, 101)
Other White	335	278515	146.6	69.9 (58.5, 83.6)	79.3 (71.5, 87.9)	83 (70, 98.4)	85.9 (76.1, 96.9)
Any Mixed Background	40	56265	211.7	101 (71.5, 142.7)	97.1 (70.4, 133.9)	109.5 (78.3, 153.2)	100.2 (72.5, 138.5)
Indian	75	65945	159	75.9 (57.1, 100.8)	100.8 (80.2, 126.8)	94.7 (71.7, 125.2)	111.4 (87.6, 141.7)
Pakistani	155	146430	195.1	93.1 (75.7, 114.4)	99.5 (84.4, 117.4)	116.6 (94.9, 143.3)	110.5 (91.9, 132.9)
Other South Asian	40	35500	183.8	87.7 (63.1, 121.8)	92.5 (67.3, 127.3)	107.6 (77.6, 149.3)	101.1 (72.9, 140.1)
African Origin	30	32160	170.2	81.2 (55.2, 119.4)	78.4 (54.1, 113.5)	97 (66.2, 142.1)	84.8 (58.3, 123.2)
Chinese	35	68685	82.4	39.3 (26.8, 57.7)	41.7 (29.9, 58.2)	49.6 (33.8, 72.8)	46.4 (32.9, 65.5)

Ethnic group	Any Amenable Death	PY at risk	Age-adjusted rates (for 100,000 PY)	Age adjusted RR and 95% CI	Age and SES adjusted RR and 95% CI	Age and UK/ROI-born adjusted RR and 95% CI	Age, UK/ROI-born and SES adjusted RR and 95% CI
FEMALES							
White Scottish	33620	22581190	148.9	100.0	100.0	100.0	100.0
Other White British	1945	1644435	103.3	69.4 (59.9, 80.3)	84.5 (80.6, 88.6)	69.9 (60.4, 80.9)	84.8 (80.9, 88.9)
White Irish	370	216905	123.5	82.9 (69.7, 98.7)	82.9 (73.4, 93.7)	82.9 (69.7, 98.7)	82.9 (73.4, 93.7)
Other White	305	319915	108.2	72.7 (61.5, 85.9)	86.1 (76.7, 96.6)	86.4 (73.1, 102.1)	94 (82, 107.7)
Any Mixed Background	30	59970	129.4	86.9 (61.2, 123.5)	83.7 (60.4, 116)	93.8 (66.4, 132.4)	86.6 (62.5, 120)
Indian	45	59925	124.6	83.7 (62.8, 111.5)	101.2 (79, 129.7)	101.6 (75.9, 136)	111.6 (85.8, 145.2)
Pakistani	85	143940	130.1	87.4 (68.5, 111.5)	90.7 (73.3, 112.3)	106 (82.5, 136.2)	100.3 (79.5, 126.6)
Other South Asian	20	28610	117.1	78.7 (51.4, 120.5)	79.7 (53.2, 119.4)	92.4 (60.3, 141.6)	86.2 (57.2, 130)
African Origin	25	28590	155.7	104.6 (69.9, 156.6)	108 (74.2, 157.1)	120.3 (80.6, 179.6)	115.7 (79.3, 168.7)
Chinese	40	68010	90.9	61.1 (44.2, 84.4)	65.8 (49.2, 87.9)	75.6 (54.2, 105.5)	73.4 (54, 99.8)

Table 5: Age adjusted rates per 100,000 population (PY) and rate ratios (RR x100) for amenable mortality (excluding IHD) by sex and ethnic group. RRs are age, socio-economic status (SES) (household tenure, combined individual and household education and Scottish Index of Multiple Deprivation SIMD) and UK/RoI-born (versus born elsewhere) adjusted, with 95% confidence intervals.

Ethnic group	Any amenable death (ex IHD)	PY at risk	Age-adjusted poisson rates (for 100,000 PY)	Age adjusted RR and 95% CI	Age and SES adjusted RR and 95% CI	Age and UK/ROI-born adjusted RR and 95% CI	Age, UK/ROI-born and SES adjusted RR and 95% CI
MALES							
White Scottish	20760	21179755	98	100.0	100.0	100.0	100.0
Other White British	1195	1571080	62.6	63.9 (54.9, 74.4)	78.7 (73.3, 84.5)	64.4 (55.3, 75)	79.1 (73.7, 84.9)
White Irish	255	202190	95.6	97.5 (75.2, 126.6)	92.8 (80.5, 107)	97.6 (75.2, 126.6)	92.8 (80.5, 107)
Other White	165	278515	71.8	73.2 (60.3, 89)	81.1 (70.1, 93.8)	90 (73.7, 109.9)	92.8 (78.3, 110)
Any Mixed Background	15	56265	71.9	73.4 (44.6, 120.7)	70.4 (43.1, 115)	80.5 (49.2, 132)	74.3 (45.4, 121.6)
Indian	30	65945	58.8	60 (39.2, 91.9)	76.4 (52.5, 111.1)	77.9 (50.8, 119.4)	90.1 (61.1, 133)
Pakistani	70	146430	83.7	85.4 (64.5, 113)	90.5 (69.4, 118.1)	110.7 (82.8, 148.1)	107.4 (80.4, 143.4)
Other South Asian	15	35500	62.7	63.9 (38, 107.6)	65.9 (39.2, 110.8)	81.4 (48.2, 137.7)	76.5 (45.1, 129.7)
African Origin	20	32160	103.8	105.9 (67.4, 166.4)	100.8 (64.5, 157.4)	130.7 (83.1, 205.6)	115 (73.2, 180.6)
Chinese	30	68685	60.9	62.1 (41.8, 92.2)	64.9 (45.3, 93.1)	81.7 (54.6, 122.3)	77.5 (53.1, 113.3)

Ethnic group	Any Amenable Death (ex IHD)	PY at risk	Age-adjusted rates (for 100,000 PY)	Age adjusted RR and 95% CI	Age and SES adjusted RR and 95% CI	Age and UK/ROI-born adjusted RR and 95% CI	Age, UK/ROI-born and SES adjusted RR and 95% CI
FEMALES							
White Scottish	23125	22581190	102.4	100	100	100	100
Other White British	1460	1644435	77.1	75.3 (67.1, 84.4)	87.2 (82.8, 91.8)	75.8 (67.5, 85)	87.5 (83.1, 92.2)
White Irish	260	216905	87.8	85.7 (73.5, 100)	86.2 (74.8, 99.4)	85.7 (73.5, 100)	86.2 (74.8, 99.4)
Other White	225	319915	78.9	77.1 (65.8, 90.3)	87.2 (76.6, 99.4)	90.2 (75.9, 107.2)	96.2 (82, 112.8)
Any Mixed Background	25	59970	94.7	92.5 (61.3, 139.4)	90 (61.2, 132.5)	99.1 (65.9, 149)	93.6 (63.6, 137.7)
Indian	30	59925	80.7	78.8 (56.6, 109.8)	90.8 (66.1, 124.7)	93.7 (66.5, 131.9)	101 (72.4, 141.1)
Pakistani	55	143940	80.6	78.7 (59.9, 103.5)	80.8 (62.9, 103.7)	93.4 (70.2, 124.4)	90 (68.4, 118.3)
Other South Asian	10	28610	67.4	65.9 (37.4, 115.9)	66.3 (38.4, 114.5)	76.4 (43.2, 135)	72.5 (41.6, 126.2)
African Origin	20	28590	123.9	121 (79.8, 183.4)	123.2 (82.9, 183.3)	137.7 (90.8, 208.7)	133.3 (89.3, 198.8)
Chinese	35	68010	74.6	72.9 (51.8, 102.6)	77 (55.5, 106.8)	88.5 (61.9, 126.5)	86.9 (61.3, 123.2)

Table 6: Age adjusted rates per 100,000 population (PY) and rate ratios (RR x100) for preventable mortality, for the population by sex and ethnic. RRs are age, socio-economic status (SES) (household tenure, combined individual and household education and Scottish Index of Multiple Deprivation SIMD) and UK/RoI-born (versus born elsewhere) adjusted, with 95% confidence intervals.

Ethnic group	Any preventable death	PY at risk	Age-adjusted poisson rates (for 100,000 PY)	Age adjusted RR and 95% CI	Age and SES adjusted RR and 95% CI	Age and UK/ROI-born adjusted RR and 95% CI	Age, UK/ROI-born and SES adjusted RR and 95% CI
MALES							
White Scottish	76165	21179755	359.6	100.0	100.0	100.0	100.0
Other White British	4275	1571080	224.1	62.3 (52.4, 74.1)	81 (77.6, 84.5)	62.8 (52.8, 74.7)	81.2 (77.8, 84.8)
White Irish	910	202190	345.3	96 (78.7, 117.1)	91.2 (82.9, 100.4)	96.1 (78.8, 117.2)	91.3 (82.9, 100.4)
Other White	575	278515	241.3	67.1 (56.9, 79.1)	77.1 (70.6, 84.2)	80.9 (69.5, 94.2)	84.8 (77, 93.4)
Any Mixed Background	85	56265	380.6	105.8 (81.5, 137.4)	102.5 (82.3, 127.7)	115 (89.5, 147.9)	106.3 (85.2, 132.7)
Indian	115	65945	228.6	63.6 (49.2, 82.2)	86.9 (71.1, 106.1)	79.7 (62.3, 102)	97 (78.9, 119.4)
Pakistani	170	146430	196	54.5 (45.2, 65.8)	58.9 (51.2, 67.8)	68.1 (56.7, 81.7)	66 (56.7, 76.9)
Other South Asian	70	35500	298.9	83.1 (61.7, 112)	87.8 (67.8, 113.7)	103.1 (77.2, 137.7)	97.3 (74.7, 126.6)
African Origin	50	32160	259.8	72.3 (51.4, 101.6)	69.4 (50.8, 94.8)	87.2 (62.4, 121.9)	76 (55.4, 104.1)
Chinese	75	68685	152.1	42.3 (32.7, 54.7)	45.9 (36.8, 57.1)	53.8 (41.8, 69.2)	51.8 (41.3, 65)

Ethnic group	Any Preventable Death	PY at risk	Age-adjusted rates (for 100,000 PY)	Age adjusted RR and 95% CI	Age and ses adjusted RR and 95% CI	Age and UK/ROI-born adjusted RR and 95% CI	Age, UK/ROI-born and ses adjusted RR and 95% CI
FEMALES							
White Scottish	51505	22581190	228.1	100.0	100.0	100.0	100.0
Other White British	2790	1644435	147.3	64.6 (54.7, 76.2)	81.6 (78.2, 85.2)	65.2 (55.3, 77)	82.1 (78.7, 85.7)
White Irish	565	216905	188	82.4 (68.4, 99.2)	82.3 (74.6, 90.7)	82.4 (68.4, 99.2)	82.3 (74.6, 90.8)
Other White	415	319915	147	64.5 (53.9, 77.1)	78.8 (70.6, 87.9)	82 (69.1, 97.4)	90.9 (80.3, 103)
Any Mixed Background	45	59970	182.9	80.2 (57.5, 111.8)	77.4 (58.2, 103)	88.9 (64.4, 122.7)	81.6 (61.3, 108.5)
Indian	50	59925	132.1	57.9 (43.3, 77.5)	73 (56.5, 94.3)	75.6 (56.7, 100.9)	85.5 (65.4, 111.7)
Pakistani	100	143940	147.1	64.5 (52.2, 79.8)	68 (56.6, 81.6)	84 (67.8, 104.1)	79.9 (65.4, 97.6)
Other South Asian	25	28610	171.4	75.2 (50.2, 112.5)	77 (51.9, 114.2)	93.9 (63, 140.2)	87.3 (58.5, 130.3)
African Origin	30	28590	186.4	81.7 (55.8, 119.6)	84.9 (60.1, 119.9)	99.4 (68.4, 144.2)	95 (67.2, 134.4)
Chinese	50	68010	119.3	52.3 (39.5, 69.2)	58.2 (46.1, 73.4)	70.5 (53.1, 93.5)	69.6 (54.3, 89.2)

Table 7: Age adjusted rates per 100,000 population (PY) and rate ratios (RR x100) for avoidable (amenable and preventable combined) mortality by sex and ethnic group. RRs are age, socio-economic status (SES) (household tenure, combined individual and household education and Scottish Index of Multiple Deprivation SIMD) and UK/RoI-born (versus born elsewhere) adjusted, with 95% confidence intervals.

Ethnic group	Any avoidable death	PY at risk	Age-adjusted rates (for 100,000 PY)	Age adjusted RR and 95% CI	Age and SES adjusted RR and 95% CI	Age and UK/ROI-born adjusted RR and 95% CI	Age, UK/ROI-born and SES adjusted RR and 95% CI
MALES							
White Scottish	90510	21179755	427.3	100.0	100.0	100.0	100.0
Other White British	5065	1571080	265.6	62.1 (52.3, 73.9)	80.6 (77.1, 84.2)	62.6 (52.6, 74.5)	80.9 (77.4, 84.5)
White Irish	1080	202190	410.4	96 (78.1, 118.2)	91.1 (84.8, 98)	96.1 (78.1, 118.3)	91.2 (84.8, 98)
Other White	685	278515	290	67.9 (57.7, 79.8)	77.8 (71.8, 84.2)	82.5 (71.1, 95.8)	86.4 (79.1, 94.3)
Any Mixed Background	100	56265	438.4	102.6 (80.2, 131.2)	99.1 (80.2, 122.4)	111.9 (88.5, 141.6)	103.2 (83.4, 127.7)
Indian	130	65945	259.5	60.7 (46.9, 78.6)	82.7 (68.2, 100.4)	77.1 (60.3, 98.5)	93.6 (76.6, 114.3)
Pakistani	215	146430	252.3	59 (49.4, 70.5)	63.8 (55.7, 73)	74.7 (63, 88.4)	72.4 (62.7, 83.7)
Other South Asian	80	35500	348.9	81.7 (62.2, 107.2)	86 (68.1, 108.7)	102.4 (78.9, 132.9)	96.3 (75.8, 122.3)
African Origin	65	32160	340.7	79.7 (59.1, 107.6)	76.4 (58, 100.5)	97.1 (72.5, 130.1)	84.4 (64, 111.4)
Chinese	90	68685	188.9	44.2 (34.4, 56.8)	47.7 (38.9, 58.6)	56.9 (44.6, 72.7)	54.6 (44.2, 67.5)

Ethnic group	Any avoidable death	PY at risk	Age-adjusted rates (for 100,000 PY)	Age adjusted RR and 95% CI	Age and SES adjusted RR and 95% CI	Age and UK/ROI-born adjusted RR and 95% CI	Age, UK/ROI-born and SES adjusted RR and 95% CI
FEMALES							
White Scottish	63200	22581190	279.9	100.0	100.0	100.0	100.0
Other White British	3415	1644435	180.8	64.6 (54.7, 76.2)	81.6 (78.4, 85)	65.2 (55.3, 76.9)	82.1 (78.8, 85.5)
White Irish	705	216905	234.1	83.6 (69.8, 100.2)	83.5 (76, 91.8)	83.6 (69.8, 100.2)	83.5 (76, 91.8)
Other White	520	319915	183.7	65.7 (55.4, 77.8)	80.3 (72.9, 88.4)	82.3 (70, 96.7)	91.1 (81.6, 101.8)
Any Mixed Background	60	59970	247.7	88.5 (65.5, 119.5)	85.3 (66.4, 109.5)	97.4 (72.9, 130.2)	89.4 (69.6, 114.7)
Indian	60	59925	161.6	57.7 (44, 75.7)	72.6 (58.1, 90.8)	74.1 (56.8, 96.7)	83.6 (66.2, 105.6)
Pakistani	125	143940	184.6	66 (53.8, 80.9)	69.4 (58.6, 82.2)	84.4 (68.9, 103.3)	80.1 (66.6, 96.2)
Other South Asian	30	28610	204.2	73 (50.9, 104.7)	74.6 (52.9, 105)	89.8 (62.9, 128.3)	83.4 (58.9, 118.1)
African Origin	40	28590	246.6	88.1 (62.8, 123.6)	91.5 (68.1, 123)	105.7 (76.1, 146.9)	101.1 (75.1, 136.1)
Chinese	70	68010	164.8	58.9 (45.1, 76.8)	65.2 (53, 80.3)	77.8 (59.6, 101.5)	76.4 (61.2, 95.5)

Table 8: Age adjusted rates per 100,000 person-years (PY) and rate ratios (RR x100) for avoidable hospitalisations by sex and ethnic group. RRs are age, socio-economic status (SES) (household tenure, combined individual and household education and Scottish Index of Multiple Deprivation SIMD) and UK/RoI-born (versus born elsewhere) adjusted, with 95% confidence intervals.

Ethnic group	Avoidable hospitalisations	PY at risk	Age-adjusted rates (for 100,000 PY)	Age adjusted RR and 95% CI	Age and SES adjusted RR and 95% CI	Age and UK/ROI-born adjusted RR and 95% CI	Age, UK/ROI-born and SES adjusted RR and 95% CI
MALES							
White Scottish	529565	12005945	4410.9	100	100	100	100
Other White British	35285	825250	4170.1	81.4 (79.8, 83)	92.7 (90.9, 94.6)	81.6 (80, 83.3)	92.9 (91, 94.8)
White Irish	6630	115335	5605	102.5 (97.9, 107.2)	100.1 (95.7, 104.7)	102.5 (98, 107.2)	100.1 (95.7, 104.7)
Other White	4740	125325	3687.3	84.2 (80.2, 88.5)	90.8 (86.5, 95.3)	91.3 (86.4, 96.6)	94.6 (89.4, 100.1)
Any Mixed Background	615	24860	2408.8	108.2 (93, 126)	107.7 (92.6, 125.2)	111.9 (96, 130.3)	109.4 (94, 127.3)
Indian	1430	32490	4296	114.2 (102.7, 127)	133.7 (120.2, 148.6)	125.5 (112, 140.6)	140.1 (125, 157)
Pakistani	3275	77290	4131.7	140.6 (131.9, 150)	145.3 (136.2, 155)	154.3 (143.8, 165.5)	152.3 (141.8, 163.5)
Bangladeshi	175	4390	3867.9	130.8 (107.1, 159.8)	136 (111.2, 166.3)	143.5 (117.1, 175.8)	142.3 (116, 174.5)
Other South Asian	365	12485	2844.1	85.9 (71.5, 103.2)	89.4 (74.5, 107.4)	93.8 (77.8, 113.1)	93.3 (77.5, 112.5)
Caribbean	85	3370	2461.4	65.1 (49.9, 84.9)	67.3 (51.5, 87.9)	69.8 (53.4, 91)	69.6 (53.2, 91)
African	225	8160	2677.6	94.3 (62.4, 142.3)	95.7 (63.4, 144.5)	103.9 (68.3, 158)	100.5 (66, 152.9)
Black Scottish or Other Black	75	2585	2828.3	98 (71, 135.5)	88.6 (63.7, 123.1)	100.8 (72.9, 139.5)	89.8 (64.5, 124.9)
Chinese	680	25395	2619.2	70.8 (62.5, 80.1)	72.3 (63.9, 81.9)	78.3 (68.8, 89.2)	76.1 (66.8, 86.7)

Ethnic group	Avoidable hospitalisations	PY at risk	Age-adjusted rates (for 100,000 PY)	Age adjusted RR and 95% CI	Age and SES adjusted RR and 95% CI	Age and UK/ROI-born adjusted RR and 95% CI	Age, UK/ROI-born and SES adjusted RR and 95% CI
FEMALES							
White Scottish	538190	13669790	3937.1	100	100	100	100
Other White British	32005	929145	3359.6	78.7 (77, 80.4)	91.7 (89.7, 93.7)	79 (77.3, 80.7)	91.7 (89.8, 93.8)
White Irish	6580	131325	4885.4	97.3 (92.8, 102.1)	97.9 (93.4, 102.7)	97.3 (92.8, 102)	97.9 (93.4, 102.7)
Other White	4855	156675	3021.7	76.8 (72.2, 81.7)	87.2 (82, 92.8)	82.5 (76.9, 88.4)	89.1 (83.1, 95.6)
Any Mixed Background	725	29455	2397.3	98.4 (86.3, 112.2)	97.4 (85.4, 111)	100.9 (88.4, 115.2)	98 (86, 111.8)
Indian	1070	31205	3347.4	107.2 (94.8, 121.2)	125.8 (111.3, 142.2)	114.8 (100.9, 130.6)	128.5 (112.9, 146.1)
Pakistani	3060	80945	3687.2	141 (129, 154.1)	146.2 (133.7, 159.8)	150.3 (136.8, 165.1)	149.2 (135.6, 164.1)
Bangladeshi	85	3580	2314	92.7 (68, 126.3)	97.1 (71.3, 132.2)	99.2 (72.7, 135.4)	99.1 (72.7, 135.2)
Other South Asian	340	11045	2993.3	102.9 (87.2, 121.6)	104.6 (88.5, 123.6)	109 (92.1, 129.1)	106.4 (89.8, 126)
Caribbean	150	4865	3047.4	96.3 (72.2, 128.4)	106.9 (80.2, 142.6)	100.9 (75.6, 134.5)	108.3 (81.2, 144.6)
African	210	7840	2600.6	94.6 (74.7, 119.9)	94.7 (74.6, 120.3)	101.8 (80.1, 129.3)	96.9 (76, 123.5)
Black Scottish or Other Black	100	2725	3616.3	116.2 (83.2, 162.2)	111.1 (80.8, 153)	119.7 (85.8, 167.1)	112.1 (81.4, 154.3)
Chinese	655	29535	2164.2	69.1 (61.7, 77.3)	74.3 (66.4, 83.2)	74.9 (66.5, 84.4)	76.3 (67.7, 85.9)

Table 9: Age adjusted rates per 100,000 population (PY) and rate ratios (RR x100) for acute avoidable hospitalisations by sex and ethnic group. RRs are age, socio-economic status (SES) (household tenure, combined individual and household education and Scottish Index of Multiple Deprivation SIMD) and UK/RoI-born (versus born elsewhere) adjusted, with 95% confidence intervals.

Ethnic group	Acute avoidable hospitalisations	PY at risk	Age-adjusted rates (for 100,000 PY)	Age adjusted RR and 95% CI	Age and SES adjusted RR and 95% CI	Age and UK/ROI-born adjusted RR and 95% CI	Age, UK/ROI-born and SES adjusted RR and 95% CI
MALES							
White Scottish	186920	12005945	1556.9	100	100	100	100
Other White British	11925	825250	1409.3	82.1 (80.3, 84)	91.5 (89.4, 93.6)	82.5 (80.7, 84.4)	91.8 (89.7, 93.9)
White Irish	2290	115335	1936.6	107 (101.5, 112.9)	105.8 (100.3, 111.6)	107.1 (101.5, 113)	105.8 (100.3, 111.6)
Other White	1690	125325	1314.6	83.5 (78.1, 89.3)	88.5 (82.7, 94.6)	93.1 (86.6, 100)	95.8 (89.1, 103)
Any Mixed Background	275	24860	1074.9	109.3 (94.3, 126.7)	109.1 (94.1, 126.5)	113.6 (97.9, 131.7)	112.2 (96.8, 130.2)
Indian	565	32490	1693.2	120.3 (100.3, 144.2)	136 (113.4, 163)	133.6 (109.9, 162.5)	147.3 (121.1, 179.2)
Pakistani	1185	77290	1492.9	123.2 (115, 132.1)	126.7 (118.2, 135.7)	136.9 (126.9, 147.7)	137.1 (127, 148.1)
Bangladeshi	70	4390	1600.5	137 (104.7, 179.4)	139.8 (107.1, 182.5)	152.7 (116.4, 200.2)	151.8 (116, 198.6)
Other South Asian	150	12485	1187.6	91.9 (73.5, 115.1)	93.9 (75.1, 117.5)	102.3 (81.4, 128.5)	101.8 (81, 127.9)
Caribbean	25	3370	752.9	52.4 (30.8, 89.1)	53.5 (31.5, 90.9)	56.4 (33.2, 95.9)	56.5 (33.2, 96)
African	95	8160	1147.6	94.2 (75, 118.4)	93.8 (74.6, 117.9)	106.2 (84.2, 133.9)	102.9 (81.6, 129.8)
Black Scottish or Other Black	35	2585	1357.6	113.7 (78.4, 164.7)	104.4 (71.9, 151.5)	118 (81.4, 170.9)	107.3 (73.9, 155.8)
Chinese	275	25395	1056	76.9 (66.7, 88.6)	78.7 (68.2, 90.7)	85.4 (73.4, 99.2)	86.3 (74.4, 100.1)

Ethnic group	Acute avoidable hospitalisations	PY at risk	Age-adjusted rates (for 100,000 PY)	Age adjusted RR and 95% CI	Age and SES adjusted RR and 95% CI	Age and UK/ROI-born adjusted RR and 95% CI	Age, UK/ROI-born and SES adjusted RR and 95% CI
FEMALES							
White Scottish	237935	13669790	1740.6	100	100	100	100
Other White British	14530	929145	1525.3	83.5 (81.7, 85.4)	92.5 (90.5, 94.6)	83.8 (82, 85.7)	92.6 (90.5, 94.7)
White Irish	2730	131325	2029	100.9 (96, 105.9)	102.4 (97.5, 107.5)	100.9 (96, 105.9)	102.8 (97.8, 108)
Other White	2155	156675	1341.5	76.6 (72.7, 80.8)	83.7 (79.4, 88.3)	81.4 (76.7, 86.4)	87.6 (82.2, 93.4)
Any Mixed Background	390	29455	1298	99.8 (88.7, 112.3)	100.3 (89.1, 112.8)	101.9 (90.5, 114.7)	107.8 (93.9, 123.8)
Indian	535	31205	1665.9	108.2 (97.3, 120.4)	120.5 (108.3, 134)	114.1 (102.3, 127.3)	127.5 (113.5, 143.2)
Pakistani	1360	80945	1641.1	116.9 (108.7, 125.7)	119.3 (110.9, 128.3)	122.6 (113.6, 132.3)	126.1 (116.4, 136.7)
Bangladeshi	55	3580	1442.1	105.1 (70.5, 156.6)	106.7 (71.7, 159)	111.2 (74.5, 165.9)	122.2 (79.4, 188)
Other South Asian	175	11045	1554	106.2 (87.6, 128.7)	107.6 (88.8, 130.4)	111.5 (91.8, 135.2)	117.3 (95, 144.9)
Caribbean	80	4865	1603.9	102.9 (75.3, 140.6)	109.3 (80, 149.5)	106.7 (78.1, 145.8)	111.6 (78.6, 158.5)
African	110	7840	1368.7	92.9 (72.8, 118.5)	92.5 (72.5, 118)	98.9 (77.3, 126.5)	98.9 (75.7, 129.2)
Black Scottish or Other Black	45	2725	1575.4	103.5 (73.6, 145.6)	100.9 (72, 141.3)	106.2 (75.6, 149.3)	110 (76.5, 158.1)
Chinese	350	29535	1152.3	75.1 (66.8, 84.3)	78.8 (70.1, 88.5)	80.2 (71.1, 90.5)	84.9 (74.6, 96.7)

Table 10: Age adjusted rates per 100,000 population (PY) and rate ratios (RR x100) for chronic avoidable hospitalisations by sex and ethnic group. RRs are age, socio-economic status (SES) (household tenure, combined individual and household education and Scottish Index of Multiple Deprivation SIMD) and UK/RoI-born (versus born elsewhere) adjusted, with 95% confidence intervals.

Ethnic group	Chronic avoidable hospitalisations	PY at risk	Age-adjusted rates (for 100,000 PY)	Age adjusted RR and 95% CI	Age and SES adjusted RR and 95% CI	Age and UK/ROI-born adjusted RR and 95% CI	Age, UK/ROI-born and SES adjusted RR and 95% CI
MALES							
White Scottish	344620	12005945	2870.4	100	100	100	100
Other White British	23495	825250	2776.7	81 (78.9, 83.1)	93.5 (91, 96)	81.2 (79.1, 83.4)	93.5 (91.1, 96.1)
White Irish	4365	115335	3690.5	100.2 (94.4, 106.3)	97.2 (91.6, 103.1)	100.2 (94.5, 106.4)	97.2 (91.6, 103.1)
Other White	3065	125325	2386.1	84.7 (79.5, 90.1)	92.2 (86.6, 98.1)	90.8 (84.4, 97.8)	93.9 (87.1, 101.2)
Any Mixed Background	340	24860	1333.9	106.8 (84.7, 134.8)	106 (84.2, 133.5)	110.2 (87.2, 139.1)	106.8 (84.7, 134.6)
Indian	870	32490	2614.8	110.5 (97.8, 124.9)	132.1 (116.9, 149.4)	120.6 (105.9, 137.3)	135.1 (118.6, 153.9)
Pakistani	2100	77290	2651.4	152.8 (139.9, 167)	158.4 (144.9, 173.1)	166.8 (151.1, 184.2)	162 (146.6, 179.2)
Bangladeshi	105	4390	2289.6	127.3 (97.2, 166.7)	134 (101.8, 176.5)	138.4 (105.3, 182)	136.9 (103.6, 180.8)
Other South Asian	215	12485	1672.1	82.4 (66.3, 102.4)	86.9 (70, 107.9)	89.1 (71.3, 111.3)	88.6 (71, 110.5)
Caribbean	60	3370	1708.5	72.5 (54, 97.5)	75.5 (56.1, 101.7)	77.4 (57.5, 104.2)	76.8 (56.9, 103.6)
African	130	8160	1542	94.6 (48, 186.3)	97.8 (49.7, 192.4)	103.1 (51.7, 205.6)	100 (50.1, 199.4)
Black Scottish or Other Black	40	2585	1470.7	86.6 (54, 138.7)	77.4 (47.9, 125.1)	88.6 (55.2, 142.2)	77.9 (48.2, 125.9)
Chinese	410	25395	1566.9	66.9 (55.8, 80.2)	68.3 (57, 81.9)	73.3 (60.8, 88.5)	70 (58, 84.5)

Ethnic group	Chronic avoidable hospitalisations	PY at risk	Age-adjusted rates (for 100,000 PY)	Age adjusted RR and 95% CI	Age and SES adjusted RR and 95% CI	Age and UK/ROI-born adjusted RR and 95% CI	Age, UK/ROI-born and SES adjusted RR and 95% CI
FEMALES							
White Scottish	301935	13669790	2208.8	100	100	100	100
Other White British	17565	929145	1844.1	75.1 (72.6, 77.6)	91.3 (88.4, 94.4)	75.4 (72.9, 77.9)	91.4 (88.4, 94.5)
White Irish	3870	131325	2873.5	94.9 (88.6, 101.7)	94.5 (88.2, 101.3)	94.9 (88.6, 101.7)	94.5 (88.2, 101.3)
Other White	2715	156675	1688.9	77 (69.8, 84.9)	90.5 (82.1, 99.8)	84.3 (75.4, 94.1)	92 (82.2, 102.9)
Any Mixed Background	335	29455	1105.9	96.9 (77.3, 121.4)	93.9 (74.9, 117.6)	100.2 (79.9, 125.8)	94.4 (75.2, 118.4)
Indian	540	31205	1687.8	106.1 (87, 129.3)	130.4 (106.9, 159)	116.8 (94.4, 144.5)	132.7 (107.2, 164.3)
Pakistani	1700	80945	2047.2	168.5 (145.7, 194.8)	176.8 (152.9, 204.5)	184.9 (157.5, 217.2)	179.9 (152.7, 211.9)
Bangladeshi	30	3580	871.3	77.2 (47.9, 124.4)	83.1 (51.4, 134.5)	84.6 (52.4, 136.5)	84.5 (52, 137.3)
Other South Asian	165	11045	1456.9	100.4 (77.6, 129.8)	102.3 (78.9, 132.5)	108.2 (83.2, 140.7)	103.6 (79.6, 135)
Caribbean	70	4865	1443.5	89.5 (56.2, 142.4)	104.7 (65.7, 166.6)	95.4 (59.8, 152.1)	105.8 (66.3, 168.8)
African	100	7840	1231.9	96.3 (65, 142.7)	98.5 (66.2, 146.5)	105.7 (70.9, 157.5)	100.2 (66.9, 150)
Black Scottish or Other Black	55	2725	2040.9	127.7 (79, 206.3)	120 (75.8, 190.1)	132.5 (81.9, 214.4)	120.8 (76.2, 191.5)
Chinese	305	29535	1014.3	63.1 (52.5, 75.9)	68.8 (57.2, 82.7)	70.3 (57.8, 85.6)	70.2 (57.6, 85.5)

Figure: Odds ratios for unplanned readmissions by ethnic group in Scotland for males and females

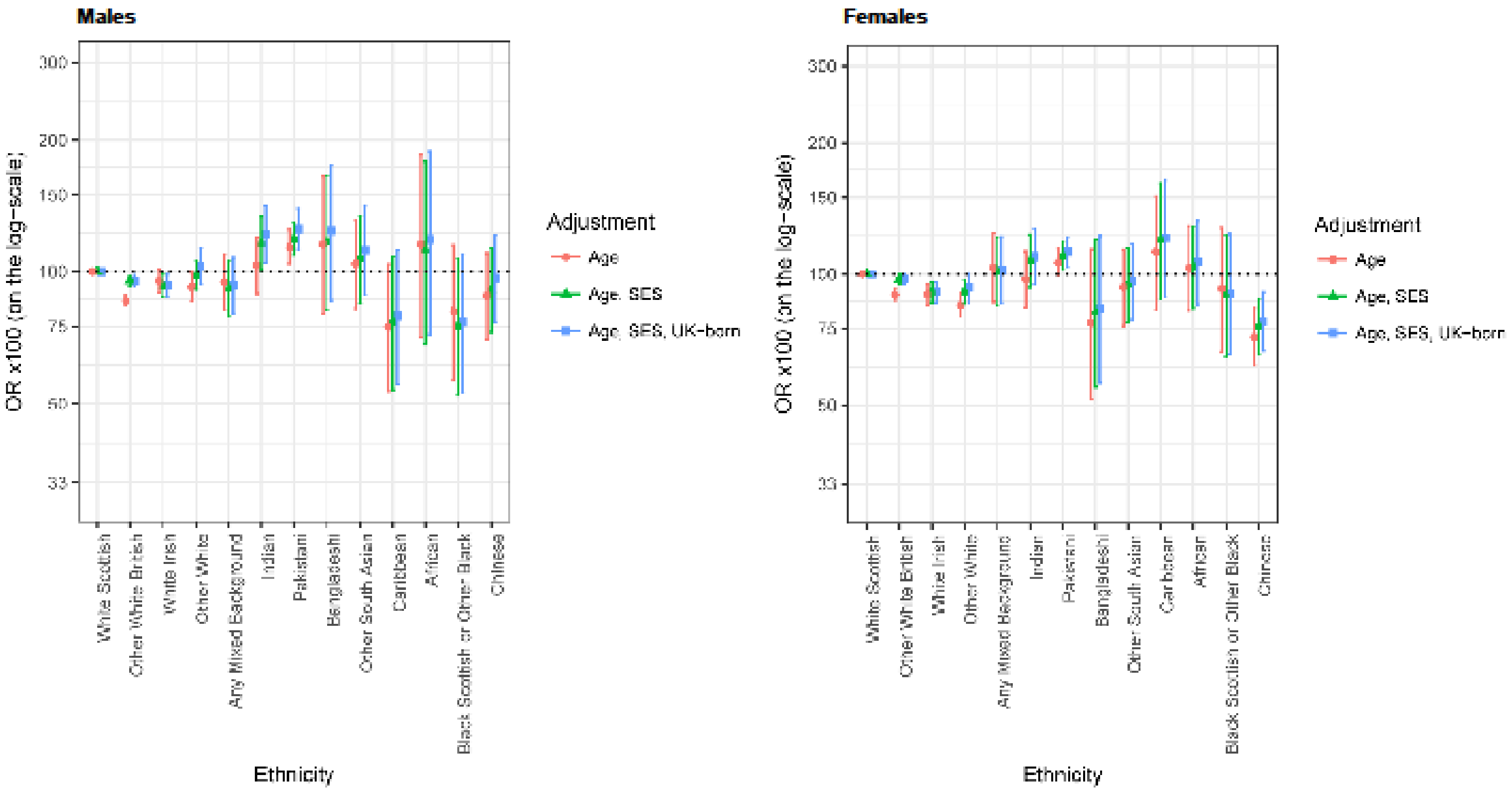

Results shown adjusted for a) age; b) age and socioeconomic status (SES); and c) age, SES and country of birth (CoB). Odds ratios (ORs) have been multiplied by 100, with the White Scottish group as the reference category.

Table 11: Number per 100,000 hospitalisations and Odds Ratios (OR x100) for unplanned readmissions by sex and ethnic group. ORs are age, socio-economic status (SES) (household tenure, combined individual and household education and Scottish Index of Multiple Deprivation SIMD) and UK/RoI-born (versus born elsewhere) adjusted, with 95% confidence intervals.

Ethnic group	Emergency readmissions	Hospitalisations	Number per 100,000	Age adjusted OR and 95% CI	Age and SES adjusted OR and 95% CI	Age and UK/ROI-born adjusted OR and 95% CI	Age, UK/ROI-born and SES adjusted OR and 95% CI
MALES							
White Scottish	269370	3750625	7182	100.0	100.0	100.0	100.0
Other White British	16570	265250	6177.5	86 (83.4, 88.7)	95.3 (92.5, 98.1)	86.4 (83.8, 89)	95.5 (92.7, 98.3)
White Irish	3150	44950	6834.2	95.2 (89.7, 101)	93 (87.7, 98.6)	95.2 (89.7, 101)	93 (87.8, 98.6)
Other White	2450	36815	6639	92.4 (85.7, 99.6)	98 (91.1, 105.4)	100.2 (91, 110.3)	103 (93.7, 113.1)
Any Mixed Background	295	4625	6810.8	94.8 (81.7, 109.9)	91.5 (79,105.8)	97.4 (83.8, 112.9)	93 (80.2, 107.5)
Indian	660	9140	7417.7	103.3 (89.1, 119.5)	116.1 (100.7, 133.4)	112.5 (95.9, 131.7)	122.2 (104.7, 142.1)
Pakistani	1555	19770	8176.2	113.8 (104, 124.6)	118.8 (108.9, 129.5)	124 (111.1, 138.1)	125.2 (112.6, 139.1)
Bangladeshi	75	930	8308.9	115.7 (79.9, 165)	117.2 (81.6, 165.4)	126.8 (87.4, 180.9)	123.9 (86.1, 175)
Other South Asian	230	3235	7478.1	104.1 (82.2, 131.2)	107 (84.9, 133.9)	112.6 (88.5, 142.4)	112.1 (88.5, 141)
Caribbean	50	940	5365.9	74.7 (53.3, 103.9)	76.7 (53.7, 108.2)	78.9 (55.9, 110.2)	79.1 (55.2, 112.1)
African	135	1740	8307.2	115.7 (70.6, 184.3)	112.1 (68.3, 178.2)	126.5 (77.2, 201.2)	118.5 (71.9, 188.7)
Black Scottish or Other Black	40	735	5827.4	81.1 (56.6, 115.1)	75.2 (52.2, 107)	83.7 (58.3, 118.9)	76.6 (53.1, 109.2)
Chinese	350	5705	6333.7	88.2 (70.2, 110.3)	91 (72.8, 113.1)	97.3 (76.9, 122.4)	96.7 (76.7, 121)

Ethnic group	Emergency readmissions	Hospitalisations	Number per 100,000	Age adjusted OR and 95% CI	Age and SES adjusted OR and 95% CI	Age and UK/ROI-born adjusted OR and 95% CI	Age, UK/ROI-born and SES adjusted OR and 95% CI
FEMALES							
White Scottish	268025	4354330	6155.3	100.0	100.0	100.0	100.0
Other White British	16440	300020	5518.5	89.7 (87.2, 92.2)	97.3 (94.6, 100)	89.9 (87.4, 92.4)	97.4 (94.7, 100.1)
White Irish	2800	49485	5526.7	89.8 (84.9, 94.9)	90.9 (86, 96)	89.8 (84.9, 94.9)	90.9 (86, 96)
Other White	2425	46575	5234.8	85 (79.7, 90.7)	91 (85.4, 96.9)	89.4 (82.6, 96.6)	93.2 (86.1, 100.7)
Any Mixed Background	400	6345	6370.8	103.5 (86.2, 123.9)	101.6 (85, 121)	105.1 (87.5, 125.9)	102.3 (85.6, 122)
Indian	500	8590	5983.3	97.2 (83.8, 112.6)	107.3 (93.1, 123.4)	101.9 (87.4, 118.5)	109.7 (94.7, 126.8)
Pakistani	1440	22710	6541.5	106.3 (98.8, 114.2)	110 (102.4, 118.1)	111 (102.4, 120.1)	112.4 (103.8, 121.7)
Bangladeshi	35	745	4749.4	77.2 (51.7, 113.9)	81.7 (55.1, 119.6)	80.7 (54.1, 119)	83.5 (56.3, 122.3)
Other South Asian	160	2890	5736.8	93.2 (76, 113.8)	94.4 (77.3, 114.7)	97 (78.9, 118.8)	96.2 (78.6, 117.2)
Caribbean	105	1575	6919.6	112.4 (82.9, 151)	119.7 (87.7, 161.6)	115.4 (84.8, 155.5)	121.2 (88.6, 163.7)
African	140	2270	6365.7	103.4 (82.6, 128.9)	104 (83.5, 128.7)	108.6 (86.5, 135.7)	106.5 (85.2, 132.3)
Black Scottish or Other Black	40	680	5701.3	92.6 (66.5, 127.8)	89.5 (64.7, 122.6)	94.5 (68, 130.2)	90.3 (65.3, 123.7)
Chinese	300	7050	4421.3	71.8 (61.7, 83.5)	75.8 (65.3, 87.8)	76.1 (65, 89.1)	78 (66.7, 90.9)

Table 12: Length of stay for malignant neoplasm of bronchus and lung (C34), chronic ischaemic heart disease (I25) and Pneumonia, organism unspecified (J18) by sex and ethnic group. Lengths of stay (derived by exponentiating logged lengths of stay) are age adjusted, with 95% confidence intervals, from linear regression models.

	**Hospitalisations C34**	**Age adjusted mean differences in length of stay (days)**	**Coefficient (x100) for age adjusted length of stay and 95% CI**	**Hospitalisations I25**	**Age adjusted mean differences in length of stay (days)**	**Coefficient (x100) for age adjusted length of stay and 95% CI**	**Hospitalisations J18**	**Age adjusted mean differences in length of stay (days)**	**Coefficient (x100) for age adjusted length of stay and 95% CI**
MALES									
White Scottish	71040	1.7672	100	71590	1.3326	100	47165	6.2332	100
Other White British	4750	1.613	84.0 (75.5, 93.4)	5615	1.3024	92.0 (86.7, 97.6)	3085	5.9786	97.7 (88.3, 108.1)
White Irish	845	1.7194	95.2 (80.5, 112.6)	895	1.3635	108.0 (90.6, 128.8)	695	6.274	100.4 (89.9, 112.1)
Other White	605	1.6232	85.1 (70.3, 103.0)	710	1.2943	89.8 (77.8, 103.8)	500	5.853	96.6 (83.5, 111.7)
Any Mixed Background	45	1.7889	102.1 (61.4, 170.0)	60	1.3417	102.4 (65.2, 160.7)	50	7.2426	108.2 (67.3, 173.9)
Indian	50	1.7692	100.2 (57.2, 175.7)	270	1.2313	72.5 (59.7, 88.0)	70	4.3996	81.0 (62.5, 104.9)
Pakistani	105	1.6138	84.1 (57.7, 122.5)	660	1.3487	104.2 (84.4, 128.5)	175	5.982	97.8 (74.2, 128.7)
Bangladeshi	.	.	.	35	1.2783	85.5 (53.4, 137.0)	10	4.6704	84.2 (40.7, 174.4)
Other South Asian	10	1.2845	44.0 (37.3, 51.8)	70	1.28	86.0 (55.5, 133.2)	30	4.4115	81.1 (57.6, 114.3)
Caribbean	10	1.8708	110.0 (60.6, 199.7)	15	1.2086	66.0 (35.5, 122.6)	.	.	.
African	.	.	.	15	1.4177	121.5 (39.1, 377.8)	15	3.6238	70.4 (49.4, 100.1)
Black Scottish or Other Black	10	3.2567	207.4 (119.7, 359.2)	10	1.5205	145.9 (76.7, 277.6)	10	9.524	123.2 (72.7, 208.6)
Chinese	80	1.9248	115.0 (81.1, 163.1)	75	1.2746	84.5 (61.9, 115.3)	55	7.0003	106.3 (80.6, 140.4)

	Hospital isations C34	Age adjusted mean differences in length of stay (days)	Coefficient (x100) for age adjusted length of stay and 95% CI	Hospi talisa tions I25	Age adjusted mean differences in length of stay (days)	Coefficient (x100) for age adjusted length of stay and 95% CI	Hospital isations J18	Age adjusted mean differences in length of stay (days)	Coefficient (x100) for age adjusted length of stay and 95% CI
FEMALES									
White Scottish	62880	1.85	100	33685	1.3036	100	48155	6.7319	100
Other White British	3130	1.9153	105.6 (92.5, 120.6)	2095	1.2673	89.4 (81.0, 98.6)	2935	5.042	84.8 (79.9, 90.1)
White Irish	760	1.8814	102.7 (87.0, 121.3)	390	1.2996	98.9 (72.7, 134.4)	665	4.8763	83.1 (74.4, 92.8)
Other White	500	2.0584	117.3 (84.8, 162.4)	290	1.2552	85.7 (64.9, 113.3)	410	5.676	91.1 (78.9, 105.0)
Any Mixed Background	15	2.9038	173.3 (111.3, 269.7)	25	1.2046	70.2 (40.6, 121.3)	65	4.1133	74.2 (54.3, 101.3)
Indian	10	1.1647	24.8 (11.3, 54.2)	70	1.2644	88.5 (36.2, 216.5)	50	3.0353	58.2 (43.5, 77.9)
Pakistani	45	1.7478	90.8 (55.7, 148.0)	175	1.2662	89.0 (67.4, 117.6)	125	4.45	78.3 (59.2, 103.6)
Bangladeshi				10	1.059	21.6 (17.0, 27.4)	.	.	.
Other South Asian	35	1.2915	41.6 (31.5, 54.8)	25	1.3775	120.8 (60.1, 243.0)	25	4.0194	73.0 (47.4, 112.2)
Caribbean				.	.	.	10	4.7323	81.5 (50.1, 132.7)
African	10	2.3265	137.2 (60.2, 312.8)	10	1.198	68.1 (18.0, 258.0)	10	12.189	131.1 (75.7, 227.2)
Black Scottish or Other Black				.	.	.	.	.	.
Chinese	95	2.862	170.9 (89.7, 325.5)	25	1.1234	43.9 (21.3, 90.3)	35	6.107	94.9 (67.9, 132.6)
